# Volumetric modulated arc therapy total body irradiation improves toxicity outcomes compared to 2D total body irradiation

**DOI:** 10.3389/fonc.2024.1459287

**Published:** 2024-09-16

**Authors:** Caressa Hui, Eric Simiele, Yuliia Lozko, Ignacio Romero, Lawrie Skinner, Michael Sargent Binkley, Richard Hoppe, Nataliya Kovalchuk, Susan M. Hiniker

**Affiliations:** ^1^ Department of Radiation Oncology, Stanford University, Stanford, CA, United States; ^2^ Department of Radiation Oncology, University of California Irvine, Irvine, CA, United States; ^3^ Department of Radiation Oncology, University of Alabama at Birmingham, Birmingham, AL, United States

**Keywords:** VMAT TBI, total body irradiation (TBI), stem cell transplant (SCT), radiation therapy, radiation toxicity, IMRT-based TBI, modern TBI

## Abstract

**Introduction:**

Volumetric modulated arc therapy (VMAT) total body irradiation (TBI) allows for greater organ sparing with improved target coverage compared to 2D-TBI. However, there is limited evidence of whether improved organ sparing translates to decreases in toxicities and how its toxicities compare to those of the 2D technique. We aimed to compare differences in toxicities among patients treated with TBI utilizing VMAT and 2D techniques.

**Methods/materials:**

A matched-pair single-institution retrospective analysis of 200 patients treated with TBI from 2014 to 2023 was performed. Overall survival (OS) and progression-free survival (PFS) were analyzed using the Kaplan–Meier method and compared using log-rank tests. Differences in characteristics and toxicities between the VMAT and 2D cohorts were compared using Fisher’s exact test.

**Results:**

Of the 200 patients analyzed, 100 underwent VMAT-TBI, and 100 underwent 2D-TBI. The median age for VMAT-TBI and 2D-TBI patients was 13.7 years and 16.2 years, respectively (*p* = 0.25). In each cohort, 53 patients were treated with myeloablative regimens (8–13.76 Gy), and 47 were treated with non-myeloablative regimens (2–4 Gy). For the entire VMAT-TBI cohort, lung Dmean, kidney Dmean, and lens Dmax were spared to 60.6% ± 5.0%, 71.0% ± 8.5%, and 90.1% ± 3.5% of prescription, respectively. For the non-myeloablative VMAT-TBI cohort, testis/ovary Dmax, brain, and thyroid Dmean were spared to 33.4% ± 7.3%, 75.4% ± 7.0%, and 76.1% ± 10.5%, respectively. For 2D-TBI, lungs were spared using partial-transmission lung blocks for myeloablative regimens. The VMAT-TBI cohort experienced significantly lower rates of any grade of pneumonitis (2% vs. 12%), nephrotoxicity (7% vs. 34%), nausea (68% vs. 81%), skin (16% vs. 35%), and graft versus host disease (GVHD) (42% vs. 62%) compared to 2D-TBI patients. For myeloablative regimen patients, rates of pneumonitis (0% vs. 17%) and nephrotoxicity (9% vs. 36%) were significantly lower with VMAT-TBI versus 2D-TBI (*p* < 0.01). Median follow-up was 14.3 months, and neither median OS nor PFS for the entire cohort was reached. In the VMAT versus 2D-TBI cohort, the 1-year OS was 86.0% versus 83.0% (*p* = 0.26), and the 1-year PFS was 86.6% and 80.0% (*p* = 0.36), respectively.

**Conclusion:**

Normal tissue sparing with VMAT-TBI compared to the 2D-TBI translated to significantly lower rates of pneumonitis, renal toxicity, nausea, skin toxicity, and GVHD in patients, while maintaining excellent disease control.

## Introduction

Total body irradiation (TBI) is an integral component of conditioning regimens for patients undergoing allogeneic hematopoietic stem cell transplantation and has been shown to improve outcomes including overall survival and lower treatment-related mortality in patients with some type of leukemia (1–5). However, despite improved oncologic outcomes with the addition of TBI, concerns over treatment-related toxicities have led to some avoidance of the use of TBI-based treatment regimens (6).

The use of TBI is associated with acute side effects including mucositis, nausea, diarrhea, and skin erythema, which may be in part related to synergistic toxicity with concurrent chemotherapies used in conditioning regimens. Long-term survivorship is associated with significant adverse treatment-related effects that may include growth impairment, endocrinopathies, pneumonitis, nephrotoxicity, cardiovascular disease, gonadal toxicity, reduced cognitive function, development of cataracts, and secondary malignancies (7–9). This has led to increased interest in improving radiation techniques to minimize toxicities without compromising oncologic outcomes.

Implementation of modern radiation techniques such as volumetric modulated arc therapy (VMAT) has been shown to be feasible and safe in a number of studies, with VMAT-TBI allowing for greater organ sparing with improved target coverage compared to 2D (10–18). However, it is unknown whether improved organ sparing translates to a decrease in clinically observed toxicities and whether these toxicities are significantly different among patients treated with VMAT-TBI compared to the 2D technique. Thus, we aimed to compare differences in toxicities and outcomes among patients treated with TBI utilizing VMAT and 2D techniques.

## Methods

### Patient cohort

In this institutional review board (IRB)-approved single-institution retrospective study, patients who received VMAT-TBI from 2019 to 2023 were identified. Patients treated with VMAT-TBI were matched with 2D-TBI patients with a 1:1 ratio based on age and total radiotherapy dose received. To minimize bias, the 2D-TBI patients were matched consecutively going back in time. Data were collected from patient medical records including demographics, disease characteristics, treatment details, outcomes, and follow-up.

### VMAT-TBI and 2D-TBI planning

Treatment planning for VMAT-TBI was described in our previous works, and only a brief summary will be given here (16, 17, 19, 20). A full-body CT scan was acquired on a Siemens Biograph™ PET-CT scanner using a 4–5-mm slice thickness. For patients taller than 115 cm, two sets of plans had to be created due to the longitudinal limitations of the treatment couch: VMAT plans with the patient positioned in the head-first supine (HFS) position and anteroposterior and posteroanterior (AP/PA) plans with the patient positioned in the feet-first supine (FFS) position. To streamline the transition between the VMAT and anteroposterior and posteroanterior (AP/PA) treatment plans, the patients were simulated on a custom rotational couch top, “Spinning Manny”. Ball bearings (BBs) were placed at the patient mid-separation, 5 cm superior to the umbilicus. Only longitudinal shifts were permitted between isocenters during treatment planning. All VMAT beams were optimized together using a dose rate of 600 MU/min. Field-in-field modulation was used to control hotspots in the AP/PA plans. After optimization, the average dose rate to fields treating lungs was kept at 100–200 MU/min to limit the average dose rate at lungs to 20 cGy/min. For the myeloablative regimen, the spared organs at risk were the lungs, kidneys, and lenses. For the non-myeloablative regimen, in addition to lungs, kidneys, and lenses, the brain, thyroid, and ovaries/testes were spared. All plans were normalized to deliver 100% of the prescription dose to 90% of the target volume while ensuring D1cc was less than 120% of the prescription dose. The entire planning process was automated using treatment planning system scripting, which decreases planning time from days to less than 4–5 hours and better standardizes plan quality as compared to manual planning (19, 20).

2D-TBI planning was performed using MU calculations based on clinical simulation measurements for an AP/PA technique at an extended Source to Skin Distance (SSD) (~608 cm), collimators at 45° and 135°, 40 × 40 cm (2) field size, and a 15-MV beam energy. The compensator layers were used to homogenize the dose distribution based on the following midline points: head, chin, neck, suprasternal notch, xiphoid, umbilicus, hip/pubis, thigh, knee, calf, and ankle. Lung blocks were generated using the lung contour contracted by 1 cm (lung-1cm) and 1 cm below the clavicle with the constant thickness of 2.5 cm of Cerrobend for every patient treated with myeloablative regimen. For patients treated with non-myeloablative regimens, lung blocks were not used. Block transmission values were verified to be 50% for a 15-MV beam using measurements in the middle of a 10-cm-thick lung slab sandwiched between two 4-cm-thick solid water slabs. For patients treated with lung blocks, electron chest wall boost fields were created and prescribed to 50% of TBI photon prescription to the depth of maximum dose. Electron energy was chosen based on the chest wall thickness measurement and ranged from 6 MeV to 20 MeV.

**Figure 1:**
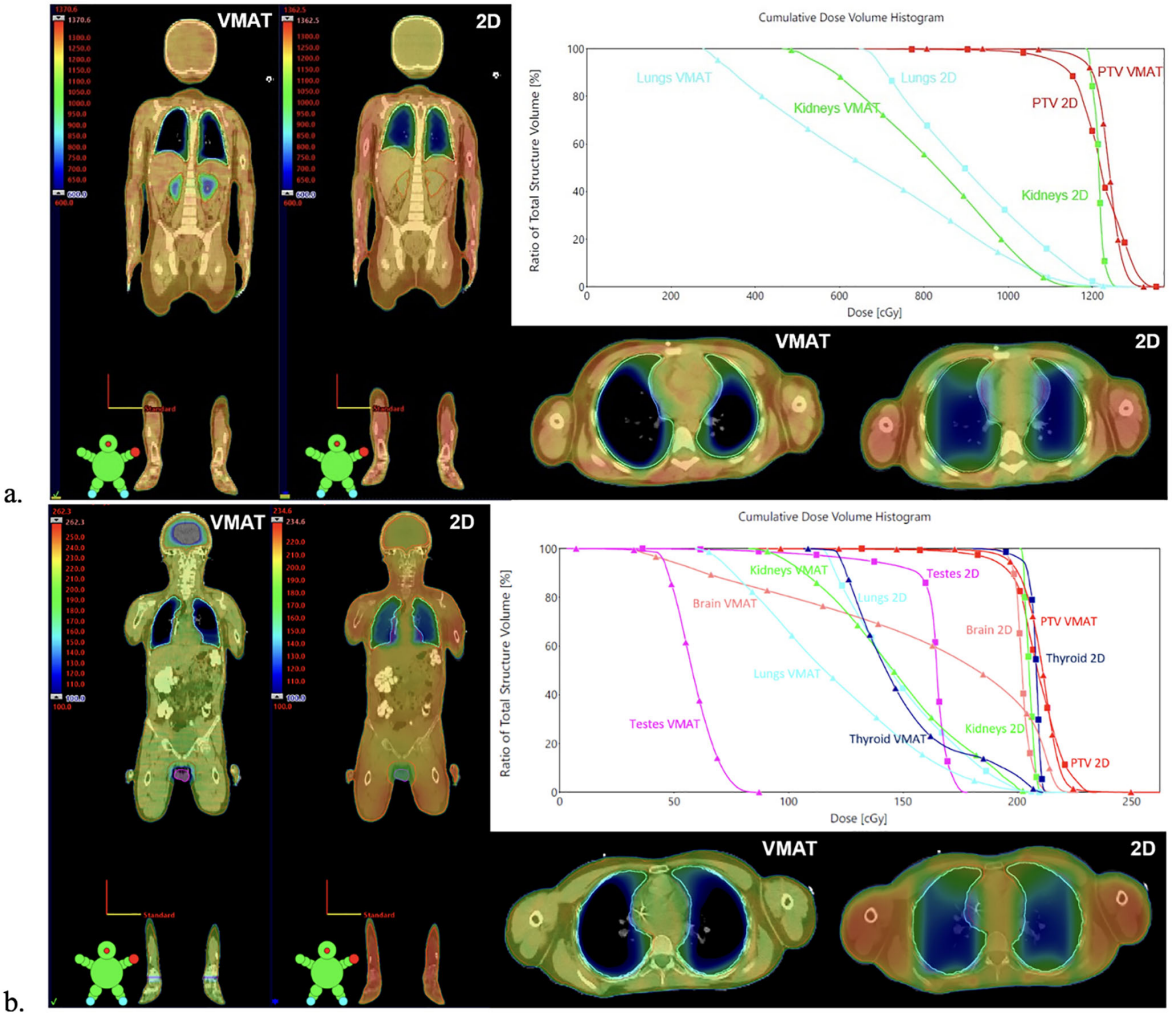
Comparison between VMAT and 2D-TBI dose distribution and DVHs for patients treated with **(A)** myeloablative regimen and **(B)** non-myeloablative regimen. 2D AP/PA plans were generated by replicating the institution’s clinical setup with the patient positioned at extended SSD with a compensator to account for differences in patient thickness, 50% transmission daily lung blocks, and electron chest-wall boosts prescribed to 50% of the photon prescription. The dose cloud is thresholded to 50% of the prescription dose. For patients treated with non-myeloablative regimens, the 2D plan also spares testes using testis block **(B)**, although it is not performed in clinical practice. VMAT, volumetric modulated arc therapy; TBI, total body irradiation; DVHs, dose–volume histograms.

**Figure 1** shows the dosimetric comparison between VMAT and 2D-TBI plans for patients treated with myeloablative and non-myeloablative regimens. For these VMAT-TBI plans, a 2D AP/PA plan was created replicating the institution’s clinical setup with the patient positioned at extended SSD with a compensator to account for differences in patient thickness, 50% transmission daily lung blocks, and electron chest-wall boosts prescribed to 50% of the photon prescription. Clinically relevant metrics were analyzed and compared between the VMAT and 2D plans on the dose–volume histograms (DVHs).

### Outcomes and statistical analysis

Time-to-event analysis was performed with the date of completion of radiation therapy as time zero, and outcomes were calculated to the date of death or censoring at the last time of contact. Overall survival (OS) was analyzed using the Kaplan–Meier method, and groups were compared using log-rank tests. Time to progression-free survival (PFS) was defined as time from the start of radiation treatment until the date of relapse or failure with death as a competing risk and patients censored at the last time of contact. Relapse was defined as biopsy-proven recurrent disease in patients with malignant diseases. For patients with non-malignant diseases, relapse was defined by blood work and bone marrow biopsy results.

Toxicity data were identified from chart review of the weekly visit notes, the patient's hospital admission notes during the peri-transplant period, and records from subsequent follow-up visits with the stem cell transplantation team. Acute toxicities were graded using Common Terminology Criteria for Adverse Events version 5.

Variables were compared using Fisher’s exact test. A *p*-value <0.05 was considered statistically significant, and all *p*-values were obtained from two-sided tests. All analyses were performed using R (version 4.2.2).

## Results

### Patient, disease, and treatment characteristics

A total of 200 patients were identified and included in the analysis. One hundred patients received VMAT-TBI, and 100 patients received 2D-TBI. The median follow-up time for the entire cohort was 14.3 months (range 1–155.7 months). The median age for patients undergoing VMAT-TBI and 2D-TBI was 13.7 years and 16.2 years, respectively. Most patients were treated for malignant diseases, and the most common disease treated overall was acute lymphoblastic leukemia (ALL) (38% and 35% for VMAT and 2D, respectively), followed by acute myeloid leukemia (AML) (15% and 18% for VMAT and 2D, respectively). There were no significant differences between patient and disease characteristics (**Table 1**). The proportions of patients undergoing a myeloablative treatment were the same in both groups, as defined by the matching process. The most commonly used myeloablative radiation treatment regimen was 12 Gy in six fractions (25% of all patients), and the most commonly used non-myeloablative radiation treatment regimen used was 2 Gy in a single fraction (30% of all patients).

**Table 1:** Patient, disease, and treatment characteristics.

	VMAT-TBI, N (%)	2D-TBI, N (%)	*p*-Value
Age at RT (median, years, range)	13.7 (0.1–64)	16.2 (1.3–57.7)	0.25
Gender			0.31
Male	60 (60%)	52 (52%)	
Female	40 (40%)	48 (48%)	
Race			0.55
Asian	23 (23%)	16 (16%)	
Black or African American	4 (4%)	4 (4%)	
Hispanic	30 (30%)	39 (39%)	
White	38 (38%)	34 (34%)	
Other	5 (5%)	3 (3%)	
ECOG PS			1.0
0-1	87 (87%)	86 (86%)	
2+	13 (13%)	14 (14%)	
Puberty			0.24
Pre	41 (41%)	32 (32%)	
Post	59 (59%)	68 (68%)	
Diagnosis/disease			0.29
Aplastic anemia	16 (16%)	23 (23%)	
MDS	2 (2%)	5 (5%)	
ALL	38 (38%)	35 (35%)	
AML	15 (15%)	18 (18%)	
Other	29 (29%)	19 (19%)	
RT dose			1.0
Non-myeloablative (2–4 Gy)	47 (47%)	47 (47%)	
Myeloablative (8-13.76 Gy)	53 (53%)	53 (53%)	

VMAT, volumetric modulated arc therapy; TBI, total body irradiation; RT, radiation therapy; ECOG PS, Eastern Cooperative Oncology Group Performance Status; MDS, myelodysplastic syndrome; ALL, acute lymphoblastic leukemia; AML, acute myeloid leukemia.

### Outcomes

**Figure 2:**
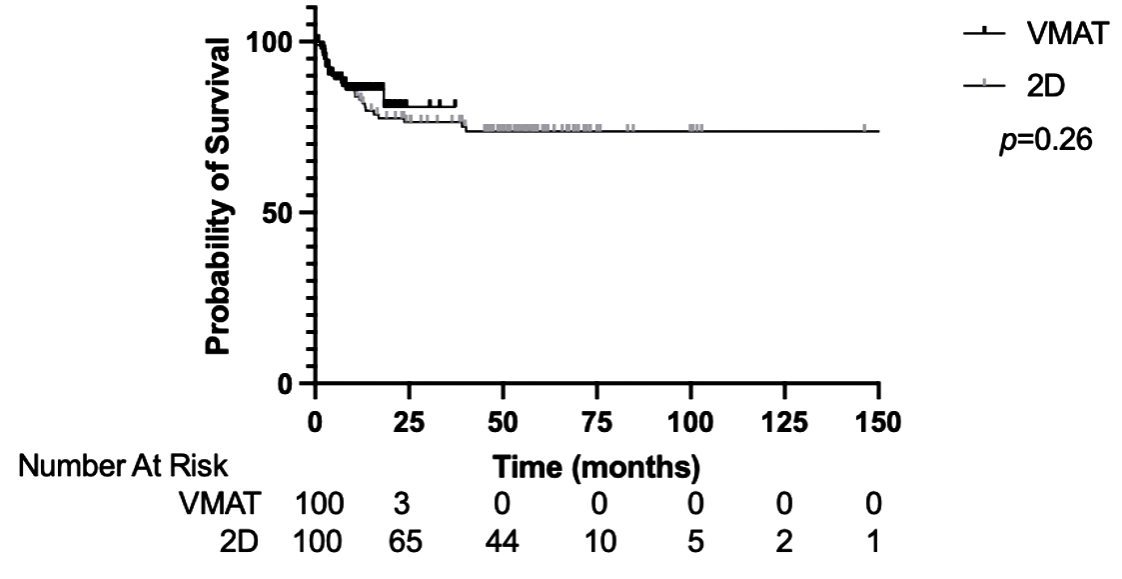
Overall survival of VMAT vs. 2D-TBI cohorts. VMAT, volumetric modulated arc therapy; TBI, total body irradiation.

The median OS for the entire cohort was not reached. The 1-year and 2-year OS was 83.9% and 77.9%, respectively (**Supplementary Figure 1**). The OS was not significantly different between the VMAT-TBI and 2D-TBI cohorts, with a 1-year OS of 86.0% and 83.0%, respectively (**Figure 2**; *p* = 0.26).

**Figure 3:**
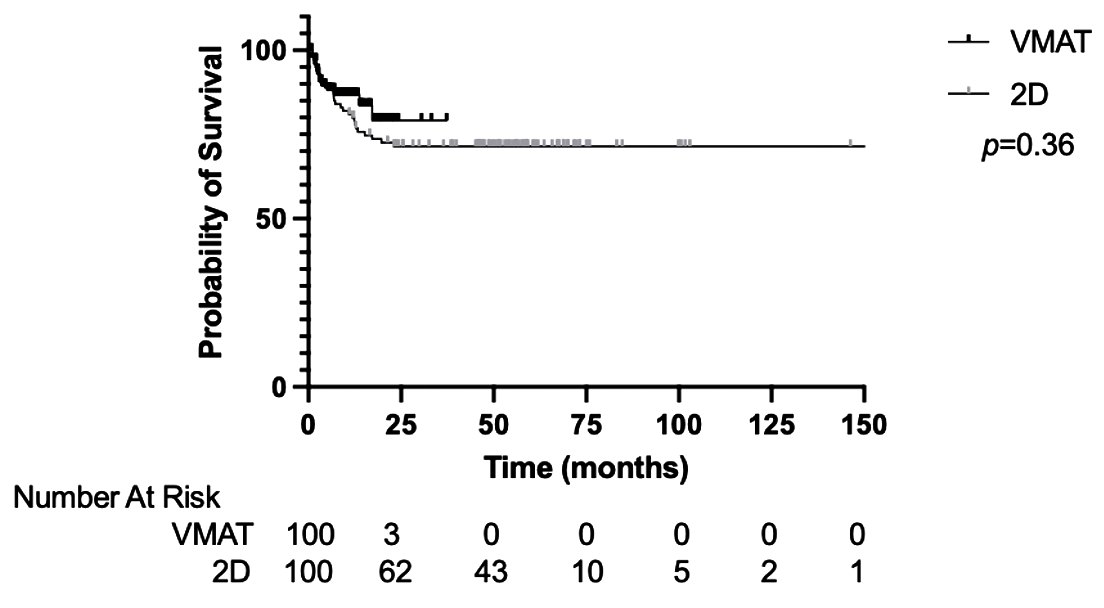
Progression-free survival of VMAT vs. 2D-TBI cohorts. VMAT, volumetric modulated arc therapy; TBI, total body irradiation.

The median PFS for the entire cohort was not reached. The 1-year and 2-year PFS was 82.4% and 73.9%, respectively (**Supplementary Figure 2**). The PFS was not significantly different between the VMAT-TBI and 2D-TBI cohorts, with a 1-year PFS of 86.6% and 80.0%, respectively (**Figure 3**; *p* = 0.36).

Four patients in the entire cohort experienced primary graft failure: two in the VMAT-TBI cohort and two in the 2D-TBI cohort. Three patients in the entire cohort experienced secondary graft failure: two in the VMAT-TBI cohort and one in the 2D-TBI cohort.

### Toxicities

In the entire cohort, the most common toxicity of any grade was mucositis (80.5%), followed by nausea (74.5%) and diarrhea (59.5%; **Table 2**). The most common grade 3+ (G3+) toxicity was mucositis (39%). The rates of any grade nausea were significantly lower in the VMAT-TBI cohort compared to the 2D-TBI cohort (68% versus 81%; *p* = 0.05). The rates of any grade of pneumonitis (2% versus 12%; *p* = 0.01) and nephrotoxicity (7% versus 34%; *p* < 0.01) were both lower for the VMAT-TBI group versus the 2D-TBI group. In the VMAT-TBI cohort, pneumonitis incidence was limited to grade 1 pneumonitis developed by two patients treated with non-myeloablative regimens, and nephrotoxicity incidence was limited to six patients with grade 1 and one patient with grade 3 toxicity. The rates of graft versus host disease (GVHD) were significantly lower for the VMAT-TBI cohort compared to the 2D cohort (42% vs. 62%, *p* < 0.01).

**Table 2:** VMAT vs. 2D-TBI toxicities for the entire cohort.

	VMAT-TBI, N (%)	2D-TBI, N (%)	*p*-Value
Diarrhea			
Any	58 (58%)	61 (61%)	0.77
Grade 1–2	57 (57%)	60 (60%)	0.77
Grade 3+	0 (0%)	1 (1%)	0.99
Fatigue			
Any	49 (49%)	63 (63%)	0.06
Grade 1–2	49 (49%)	63 (63%)	0.06
Grade 3+	0 (0%)	0 (0%)	1.0
Nausea			
Any	68 (68%)	81 (81%)	0.05*
Grade 1–2	66 (66%)	80 (80%)	0.038*
Grade 3+	2 (2%)	1 (1%)	1.0
Mucositis			
Any	84 (84%)	77 (77%)	0.28
1–2	47 (47%)	36 (36%)	0.15
3+	37 (37%)	41 (41%)	0.66
Pneumonitis			
Any	2 (2%)	12 (12%)	0.01*
1–2	2 (2%)	9 (9%)	0.058*
3+	0 (0%)	3 (3%)	0.25
Nephrotoxicity			
Any	7 (7%)	34 (34%)	<0.01*
1–2	6 (6%)	30 (30%)	<0.01*
3+	1 (1%)	4 (4%)	0.37
Skin			
Any	16 (16%)	35 (35%)	<0.01*
1–2	16 (16%)	35 (35%)	<0.01*
3+	0 (0%)	0 (0%)	1.0
GVHD			
Any	42 (42%)	62 (62%)	<0.01*
1–2	34 (34%)	51 (51%)	0.02*
3+	8 (8%)	11 (11%)	0.63

VMAT, volumetric modulated arc therapy; TBI, total body irradiation; GVHD, graft versus host disease.* denotes statistical significance.

Fifty-three patients from each cohort received a myeloablative regimen (dose range, 8–13.76 Gy). In this group of patients who underwent a myeloablative regimen, the most common toxicity of any grade was mucositis (95.3%), followed by nausea (87.0%), fatigue (67%), and diarrhea (66%; **Table 3**). The most common grade 3+ toxicity was mucositis (58.5%). The rate of any pneumonitis was significantly lower in the VMAT-TBI group compared to the 2D group (0% versus 17%, *p* < 0.01). The rates of any nephrotoxicity were also significantly lower in the VMAT-TBI cohort when compared to the 2D-TBI cohort (9% versus 36%; *p* < 0.01).

**Table 3:** VMAT vs. 2D-TBI toxicities for myeloablative regimens.

	VMAT-TBI N = 53 (N, %)	2D N = 53 (N, %)	*p*-Value
Diarrhea			
Any	35 (66%)	35 (66%)	1.0
Grade 1–2	35 (66%)	35 (66%)	1.0
Grade 3+	0 (0%)	1 (2%)	0.99
Fatigue			
Any	29 (55%)	42 (79%)	<0.01*
Grade 1–2	29 (55%)	42 (79%)	<0.01*
Grade 3+	0 (0%)	0 (0%)	1.0
Nausea			
Any	44 (83%)	48 (91%)	0.39
Grade 1–2	43 (81%)	48 (91%)	0.26
Grade 3+	1 (2%)	0 (0%)	0.99
Mucositis			
Any	50 (94%)	51 (96%)	0.99
1–2	24 (45%)	15 (28%)	0.11
3+	26 (49%)	36 (68%)	0.075
Pneumonitis			
Any	0 (0%)	9 (17%)	<0.01*
1–2	0 (0%)	6 (11%)	0.026*
3+	0 (0%)	3 (6%)	0.024*
Nephrotoxicity			
Any	5 (9%)	19 (36%)	<0.01*
1–2	4 (8%)	15 (28%)	0.01*
3+	1 (2%)	4 (8%)	0.05*
Skin			
Any	12 (23%)	18 (34%)	0.28
1–2	12 (23%)	18 (34%)	0.28
3+	0 (0%)	0 (0%)	1.0
GVHD			
Any	27 (51%)	37 (70%)	0.07
1–2	24 (45%)	27 (51%)	0.69
3+	3 (6%)	10 (19%)	0.07

VMAT, volumetric modulated arc therapy; TBI, total body irradiation; GVHD, graft versus host disease.* denotes statistical significance.

All the cases of grade 3+ nephrotoxicity were in patients who underwent a myeloablative treatment regimen. Only one patient developed G3+ nephrotoxicity in the VMAT-TBI cohort. No patients who underwent VMAT-TBI experienced any grade 3+ pneumonitis. Of the three patients who experienced grade 3+ pneumonitis in the 2D-TBI group, all of them underwent a myeloablative treatment regimen.

### Dosimetry

The relevant average achieved DVH metrics for the VMAT-TBI cohort are shown in **Table 4**. The average patient height was 149 ± 29.6 cm (range, 83.6–197.3 cm), and the average maximum patient width was 41.7 ± 8.9 cm (range, 24.9–60.3 cm). The volume of the target receiving prescription dose was equal to 90% for all cases except for one patient where 85% of the target volume received the prescription dose. The average D1cc to the target was 120.7% ± 6.3%. Lung and lung-1cm average mean doses were 60.6% ± 5.0% and 44.4% ± 6.7% of prescriptions, respectively. The average kidney mean dose was 71% ± 8.5% of the prescription dose. For the patients receiving non-myeloablative doses, the average mean doses to the brain, thyroid, and ovaries/testes were 75.4% ± 7.0%, 76.1% ± 10.5%, and 33.4% ± 7.3% of prescription, respectively.

**Table 4:** Plan quality metrics achieved for 100 patients treated with VMAT-TBI.

		Constraint	Average (% of Rx)	σ
**Myelo- and non-myeloablative regimen**	PTV	D1cc < 120%	120.7%	6.3%
	Lungs	Dmean < 55%	60.6%	5.0%
	Lung-1cm	Dmean < 40%	44.4%	6.7%
	Kidneys	Dmean < 75%	71.0%	8.5%
	Lenses	Dmax < 90%	90.1%	3.5%
**Non-myeloablative regimen**	Testes/ovaries	Dmax < 30%	33.4%	7.3%
	Brain	Dmean < 75%	75.4%	7.0%
	Thyroid	Dmean < 75%	76.1%	10.5%

VMAT, volumetric modulated arc therapy; TBI, total body irradiation.

In the 2D-TBI cohort, all organs received close to the prescription dose except for lungs that were spared using 50% transmission daily lung blocks for patients treated with myeloablative regimens. Chest wall boosts to an additional 6 Gy in two fractions were delivered to reach full coverage of the ribs and chest wall behind the lung blocks. From the dosimetric analysis based on 10 patients with simulated 2D-TBI plans, the mean lung dose was on average 80% of prescriptions compared to 55.1% of prescriptions with VMAT-TBI (16, 19). The VMAT technique also enabled a decrease of dose to other organs [kidney Dmean (−32.5%) and lens Dmax (−5.3%)], and in addition, for 2 Gy prescription: testes/ovaries Dmean (−41.5%), brain Dmean (−22.6%), and thyroid Dmean (−18.2%). In addition, VMAT-TBI enabled statistically significant improvement in average Planning Target Volume (PTV) D90% coverage (100% with VMAT-TBI and 92.9% with 2D-TBI, *p* < 0.001) and average PTV Dmean dose (103.9% with VMAT and 100.7% with 2D-TBI, *p* < 0.001). The average PTV Dmax and Dmin did not have statistically significant differences between VMAT-TBI and 2D-TBI techniques (118.8% vs. 116.8% and 54.7% vs. 55.1%, respectively). The average PTV V110% was also similar between techniques, at 1.6% with VMAT-TBI and 1.5% with 2D-TBI (*p* = 0.44).

## Discussion

This single-institution retrospective study is the largest series comparing the outcomes and toxicities between patients treated with VMAT-TBI and 2D-TBI and builds upon our previously published experience on early outcomes and toxicities of the first patients treated with VMAT-TBI at our institution (10). In this cohort of patients who underwent VMAT-TBI matched to patients who underwent 2D-TBI, VMAT-TBI resulted in a more favorable toxicity profile with excellent oncologic outcomes. The normal tissue sparing with VMAT-TBI compared to the 2D-TBI translated to significantly lower rates of pneumonitis, renal toxicity, nausea, skin toxicity, and GVHD in patients while maintaining excellent disease control.

In our study, no patients treated with VMAT-TBI experienced grade 3+ pneumonitis, which compares favorably not only to our matched 2D-TBI cohort but also to historically reported high rates of pulmonary toxicity of up to 71% with 2D-TBI treatments (21). For myeloablative treatments, 50% of patients in our 2D-TBI cohort experienced some type of pulmonary toxicity compared to 0% in the VMAT-TBI cohort. Our results showing low rates of pneumonitis are supported by previously published single-institutional experiences using VMAT-TBI (11–15). Tas et al. reported outcomes for 30 patients with AML or ALL treated with VMAT-based TBI and found no incidence of grade 3+ toxicities (12). In a series of 29 patients treated with VMAT-TBI using myeloablative regimens, Melton et al. found only one instance of pneumonitis (15). In another retrospective study that included 44 patients (32 myeloablative and 14 non-myeloablative), Zhang-Velten et al. reported 9% of grade 3+ pneumonitis, but of the four cases, only one (2%) was deemed to be likely attributable to radiation therapy alone (13). A study by Ladbury et al. performed a matched-pair retrospective review of tomotherapy-based TBI versus 2D-TBI and found no rates of pneumonitis in the tomotherapy cohort and 19.2% of pneumonitis in the 2D-TBI cohort (18), similar to our findings in patients treated with myeloablative regimen (0% in VMAT-TBI cohort vs. 17% in 2D-TBI cohort, *p* < 0.01).

In our study, the rates of nephrotoxicity of any grade were also low in the VMAT-TBI cohort and compared favorably to the 2D cohort: 7% in the VMAT-TBI cohort and 31% in the 2D-TBI cohort. Out of seven patients who developed nephrotoxicity in the VMAT-TBI cohort, six of them developed grade 1 toxicity. Grade 3+ nephrotoxicity was observed in one patient (1%) in the VMAT-TBI cohort and four patients (4%) in the 2D-TBI cohort, which compared favorably with previously published reports. Although most VMAT-TBI studies report no nephritis attributable to radiation, there are a few studies that report varying rates of nephrotoxicity rates including acute kidney injury and nephritis (13%–28%) (13, 15). The series by Melton et al., who treated using VMAT-TBI, reported a 14% rate of grade 3+ acute kidney injury, while the study by Zhang-Velten et al. reported a 13% rate of grade 3+ nephrotoxicity (13, 15). These studies reported no sparing of kidneys, whereas, in our study, we spared kidneys to a mean dose of 71% of the prescription. This dosimetric limit is motivated by Lawton et al. showing that kidney toxicity decreased from 29% vs. 0% when 30% kidney shielding was used (22).

Mucositis was the most common G3+ toxicity in our study: 37% in the VMAT-TBI cohort compared to 41% in the 2D-TBI cohort. Mucositis rates vary greatly in the literature on VMAT or tomotherapy-based TBI: 100% (11), 71% (13), 55% (14), and 34%–39.5% (10, 15). Differences in the rate of reported mucositis in VMAT-TBI studies may be attributable to differences in radiotherapy prescription, limiting dose heterogeneity during planning, mucositis reporting criteria, concurrent conditioning systemic therapy regimens, etc. In our study, the plan global maximum was limited to 120% of prescriptions, and the dose within the oral cavity was limited to 110% of prescriptions. Unfortunately, we found that changing the technique from 2D-TBI to VMAT-TBI did not significantly improve the mucositis rates. In addition, we observed G3+ mucositis in 16 patients undergoing low-dose VMAT or 2D-TBI, indicating that mucositis is likely multifactorial in the settings of concomitant chemotherapy, GVHD prophylaxis, and transplant toxicity not fully attributable to TBI. Keit et al. reported two deaths from G5 oral mucositis after VMAT-TBI, which led to the institution lowering the oropharyngeal mucosa mean dose to less than 6.9 Gy (14). Although no further deaths from mucositis were observed by Keit et al., lowering the dose to the oral cavity did not reduce G3+ mucositis rates.

In addition to lower lung and kidney toxicity, patients treated with VMAT-TBI compared to 2D-TBI experienced lower nausea, skin, and GVHD toxicity. The lower rates of nausea seen in the VMAT-TBI cohort may be due to limiting hotspots to the bowel with VMAT-TBI, more accurate dose calculation, better image guidance compared to 2D-TBI, and improved patient comfort when lying down during the delivery of VMAT-TBI. The rate of any GVHD in the VMAT-TBI cohort was 42%, which was significantly lower than the 62% seen in the 2D-TBI cohort. Consistent with our findings, Ladbury et al. reported reduced grade 2–4 GVHD for patients treated with tomotherapy-based TBI compared to 2D-TBI (41.7% vs. 79.2%, *p* = 0.02) (18). The incidence of grade 3+ GVHD in our study for patients undergoing VMAT-TBI was 8%, with no deaths due to GVHD. There have been previously published data suggesting that a TBI-containing regimen is a significant risk factor for GVHD (23–25). It has been hypothesized that TBI may cause an increased release of more inflammatory cytokines that result in increased endothelial cell damage that precipitates a cytokine storm that is associated with GVHD (26, 27). Although the exact mechanisms are unknown, the importance of reducing the risk of GVHD is paramount, as a few series using tomotherapy-based TBI have reported deaths due to GVHD (28–30).

There are several limitations to our single-institution retrospective study. A key limitation is our heterogeneous patient cohort, which includes multiple disease types treated, radiation treatment doses, and fractionation schemes. Although there was a significant difference in the doses and fractionations used between the two cohorts, the proportion of patients treated with a myeloablative versus non-myeloablative radiation regimen was the same, as our matching criteria were based on whether patients received a myeloablative regimen. Another limitation of our study is that we included patients treated over the span of a decade in the 2D-TBI cohort in order to find matched patients, while the VMAT-TBI cohort is more modern since this modality was adopted more recently. However, no changes to the 2D-TBI technique were found during this time. Finally, reporting of adverse events such as pneumonitis and nephrotoxicity is not standardized in the existing published TBI literature. The lack of standardization in reporting criteria may account for differences seen between the toxicities that were found in this study and others, as well as the difficulty of attributing toxicities to TBI alone. We reported no nephrotoxicity for cases that were clearly not due to TBI such as acute kidney injuries from medications with timing not consistent with radiation-induced nephrotoxicity. In efforts to help standardize pneumotoxicity reporting, we utilized criteria from the Vogel et al. review paper focused on pulmonary toxicities after TBI (21).

Additionally, our team initiated the workflow automation and implementation of total marrow irradiation (TMI) and total marrow and lymphoid irradiation (TMLI) in our clinic. TMI and TMLI represent novel approaches to the targeted delivery of large-field radiotherapy in hematopoietic stem cell transplantation (HSCT) conditioning. TMI/TMLI not only focuses specifically on the marrow or the marrow and lymphoid system, allowing for further improvement in sparing of organs at risk and dose escalation, but also addresses a new patient population, including those with relapsed or refractory disease ineligible for standard transplant regimens, older patients over 60, those with comorbidities that preclude myeloablative TBI, patients with haplo-identical donors, and those needing larger fraction sizes or treatment for multiple myeloma (31–33). Further studies on the use of both VMAT-TBI, TMI, and TMLI techniques and the inclusion of these techniques in large, ongoing clinical trials with standardized toxicity data collection are necessary to validate our findings. Further prospective data are necessary to help develop standardized dose constraints for organs at risk, and collaboration among institutions, professional organizations, and research groups can help standardize TBI protocols and may lead to new insights, addressing gaps in clinical knowledge.

## Conclusion

VMAT-TBI offers improved organ sparing when compared to 2D-TBI. Lower doses to organs at risk translated to significantly lower rates of pneumonitis, renal toxicities, nausea, skin toxicities, and GVHD in patients without compromising oncologic outcomes. Longer-term follow-ups are necessary to further evaluate late toxicities. Implementation of VMAT-TBI into clinical trials should be considered to minimize toxicities for patients undergoing conditioning regimens.

## Data Availability

The raw data supporting the conclusions of this article will be made available by the authors, without undue reservation.

## Supplementary material

Supplementary Figure 1 Overall Survival of the Entire Cohort.

Supplementary Figure 2 Progression Free Survival of the Entire Cohort.
