# Supplementary figures and images for: Volumetric modulated arc therapy total body irradiation improves toxicity outcomes compared to 2D total body irradiation
Source: Front Oncol. 2024 Sep 16;14:1459287. doi: 10.3389/fonc.2024.1459287 (PMC11439880; doi:10.3389/fonc.2024.1459287)

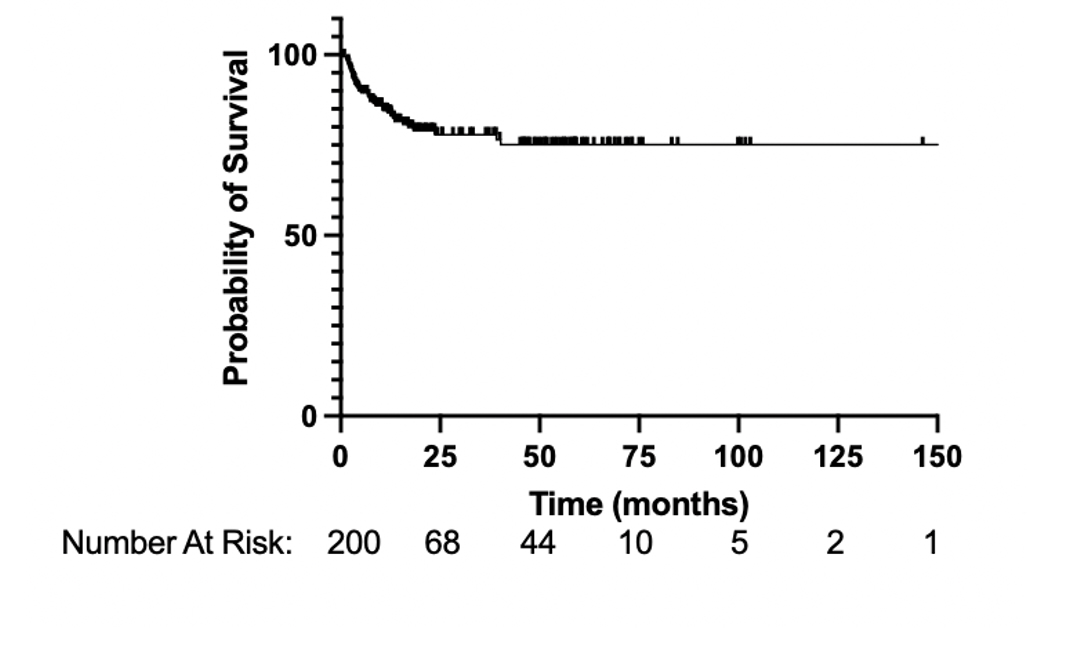

Supplement: Supplementary Figure 1 — Overall Survival of the Entire Cohort. [file Image1.jpeg]

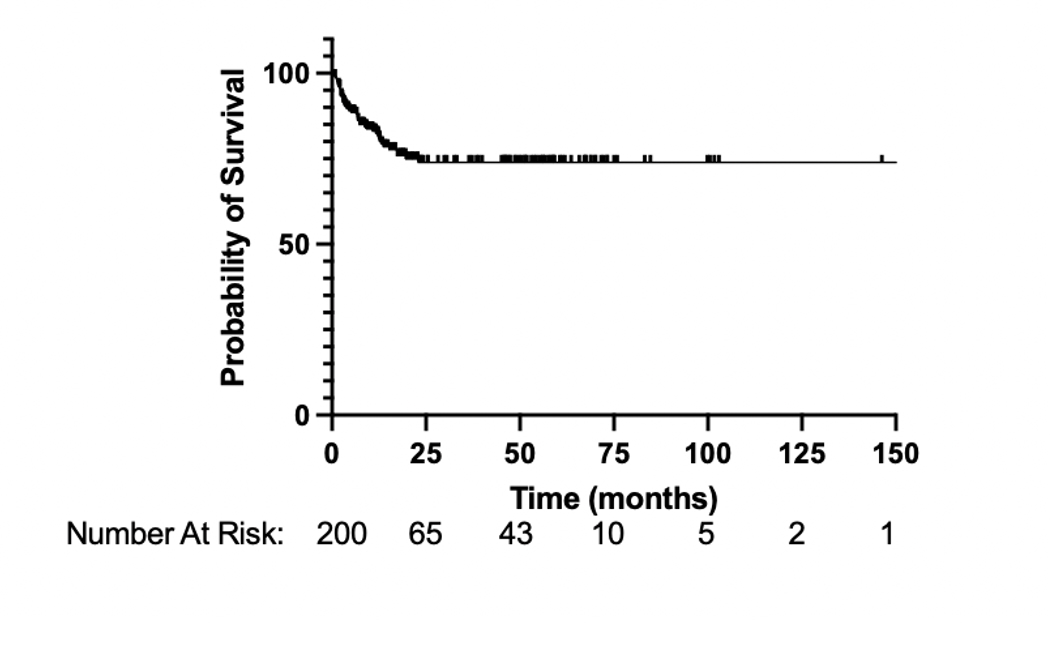

Supplement: Supplementary Figure 2 — Progression Free Survival of the Entire Cohort. [file Image2.jpeg]
